# Supplementary material for: Mutations in PCYT2 disrupt etherlipid biosynthesis and cause a complex hereditary spastic paraplegia
Source: Brain. 2019 Oct 22;142(11):3382–97. doi: 10.1093/brain/awz291 (PMC6821184; doi:10.1093/brain/awz291)
Supplement: awz291_Supplementary_Data [file awz291_supplementary_data.zip › awz291-Suppl_data/Supplementary_Data4.zip!/Supplementary information 4 - plasma lipidomics.zip!/Plasma lipidomics/Multiple comparison/Heatmap VIP.pdf]

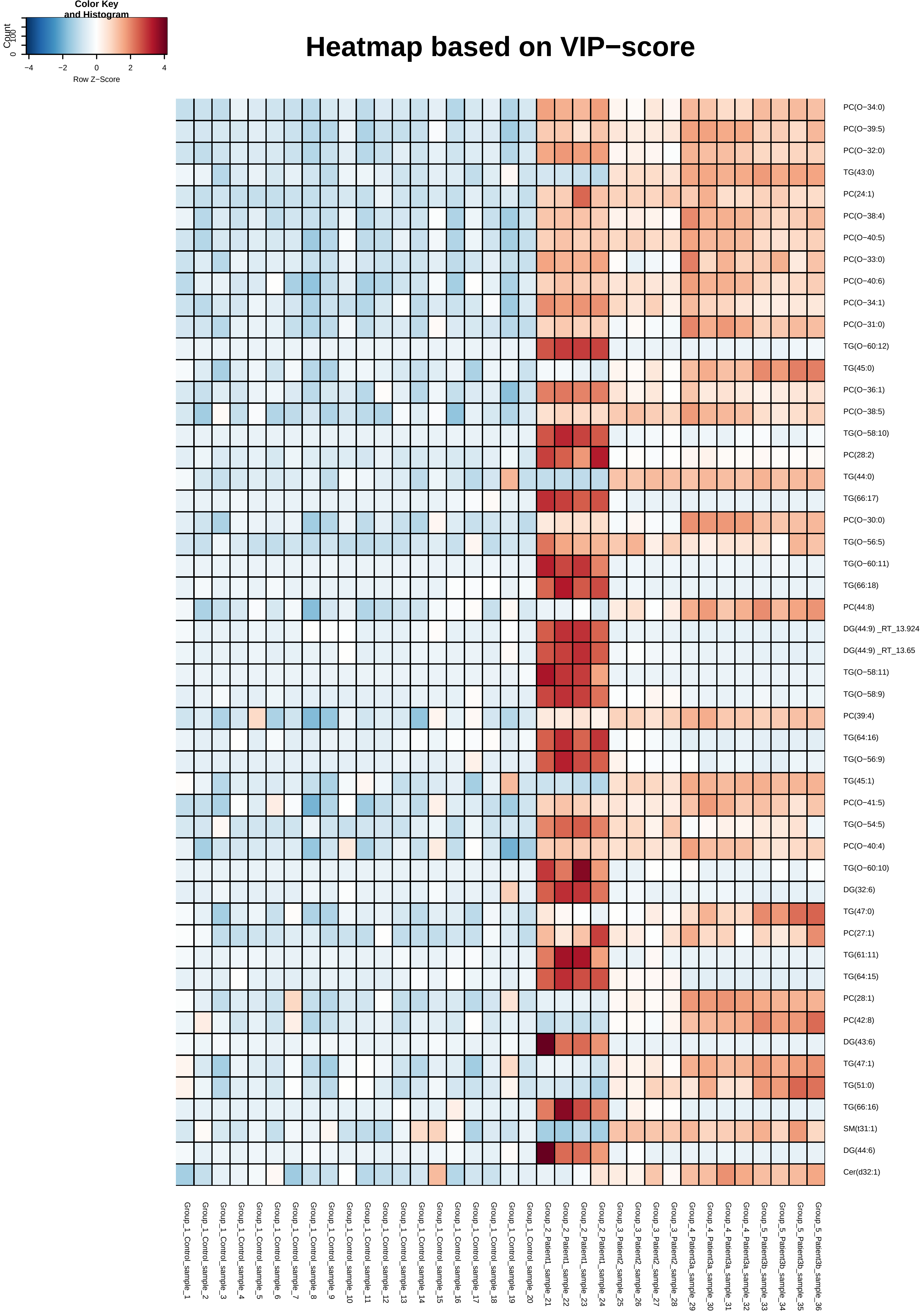
Color Key
and Histogram
Count
0
100
−4
−2
0
2
4
Row Z−Score
Heatmap based on VIP−score
PC(O−34:0)
PC(O−39:5)
PC(O−32:0)
TG(43:0)
PC(24:1)
PC(O−38:4)
PC(O−40:5)
PC(O−33:0)
PC(O−40:6)
PC(O−34:1)
PC(O−31:0)
TG(O−60:12)
TG(45:0)
PC(O−36:1)
PC(O−38:5)
TG(O−58:10)
PC(28:2)
TG(44:0)
TG(66:17)
PC(O−30:0)
TG(O−56:5)
TG(O−60:11)
TG(66:18)
PC(44:8)
DG(44:9) _RT_13.924
DG(44:9) _RT_13.65
TG(O−58:11)
TG(O−58:9)
PC(39:4)
TG(64:16)
TG(O−56:9)
TG(45:1)
PC(O−41:5)
TG(O−54:5)
PC(O−40:4)
TG(O−60:10)
DG(32:6)
TG(47:0)
PC(27:1)
TG(61:11)
TG(64:15)
PC(28:1)
PC(42:8)
DG(43:6)
TG(47:1)
TG(51:0)
TG(66:16)
SM(t31:1)
DG(44:6)
Cer(d32:1)
Group_1_Control_sample_1
Group_1_Control_sample_2
Group_1_Control_sample_3
Group_1_Control_sample_4
Group_1_Control_sample_5
Group_1_Control_sample_6
Group_1_Control_sample_7
Group_1_Control_sample_8
Group_1_Control_sample_9
Group_1_Control_sample_10
Group_1_Control_sample_11
Group_1_Control_sample_12
Group_1_Control_sample_13
Group_1_Control_sample_14
Group_1_Control_sample_15
Group_1_Control_sample_16
Group_1_Control_sample_17
Group_1_Control_sample_18
Group_1_Control_sample_19
Group_1_Control_sample_20
Group_2_Patient1_sample_21
Group_2_Patient1_sample_22
Group_2_Patient1_sample_23
Group_2_Patient1_sample_24
Group_3_Patient2_sample_25
Group_3_Patient2_sample_26
Group_3_Patient2_sample_27
Group_3_Patient2_sample_28
Group_4_Patient3a_sample_29
Group_4_Patient3a_sample_30
Group_4_Patient3a_sample_31
Group_4_Patient3a_sample_32
Group_5_Patient3b_sample_33
Group_5_Patient3b_sample_34
Group_5_Patient3b_sample_35
Group_5_Patient3b_sample_36
